# An Improved Piecewise Linear Chaotic Map Based Image Encryption Algorithm

**DOI:** 10.1155/2014/275818

**Published:** 2014-01-23

**Authors:** Yuping Hu, Congxu Zhu, Zhijian Wang

**Affiliations:** ^1^School of Information, Guangdong University of Finance & Economics, Guangzhou 510320, China; ^2^School of Information Science and Engineering, Central South University, Changsha 410083, China

## Abstract

An image encryption algorithm based on improved piecewise linear chaotic map (MPWLCM) model was proposed. The algorithm uses the MPWLCM to permute and diffuse plain image simultaneously. Due to the sensitivity to initial key values, system parameters, and ergodicity in chaotic system, two pseudorandom sequences are designed and used in the processes of permutation and diffusion. The order of processing pixels is not in accordance with the index of pixels, but it is from beginning or end alternately. The cipher feedback was introduced in diffusion process. Test results and security analysis show that not only the scheme can achieve good encryption results but also its key space is large enough to resist against brute attack.

## 1. Introduction

With the rapid development of network communication technology, multimedia information such as digital images are more commonly and frequently transmitted in public communication network. Therefore, it is particularly important to protect images from piracy. As a result, image encryption technology becomes an important issue of cryptography. Image data have two features, namely, bulky data capacity and strong correlations among adjacent pixels. For this reason conventional cipher algorithms are not directly suitable for image encryption. Chaotic cryptography then was drawn attention by researchers due to its many good properties, such as ergodicity, sensitive dependence on initial conditions, random-like behavior, and high efficiency in image encryption.

In recent years, many image encryption algorithms have been proposed [1–11]. The confusion and diffusion processes in cryptography proposed by Shannon [12] are applied in image encryption successfully. These processes include a permutation-diffusion structure, while many proposed chaotic image encryption systems [8–11] adopted Arnold cat map to shuffle the positions of the pixels by confuse phase, and the permutation process was separated from diffusion process. Arnold cat map has some weaknesses [13]. One is that the periodic states appear in very limited iteration times. Another one is that the width and height of the processed image must be equal, or the image cannot be permuted directly. What is more, each pixel position of different images in same size is fixed after the diffuse phase. Thus it is not suitable for the practical application in chaotic cryptography. If the permutation process was separated from diffusion process, efficiency will be reduced. In [14], Wang and Jin proposed an image encryption algorithm. They confuse the plain image by using logistic map and Game of Life instead of Arnold cat map firstly. Then they use piecewise linear chaotic map (PWLCM) to diffuse each pixel of the image. In this paper, we firstly proposed a improved piecewise linear chaotic map (MPWLCM) model. Then we use the MPWLCM to shuffle positions and diffuse values of pixels in plain image simultaneously. Test results and security analysis not only show that the scheme can achieve good encryption result but also show that the key space is large enough to resist against brute attack.

## 2. The MPWLCM Map

The PWLCM can be described as

(1) $$\begin{array}{c} x_{n+1}=F\left(x_{n},p\right)=\{\begin{array}{cc} \frac{x_{n}}{q}, & x_{n}\in \left[0,q\right)\\ \frac{x_{n}-q}{0.5-q}, & x_{n}\in \left[q,0.5\right)\\ F\left(1-x_{n},q\right), & x_{n}\in \left(0.5,1\right), \end{array} \end{array}$$

where *x*_*n*_ ∈ (0,1), when control parameter *q* ∈ (0,0.5), (1) evolves into a chaotic state [14], and *q* can be served as a secret key. PWLCM system has uniform invariant distribution and very good ergodicity, confusion, and determinacy, so it can provide excellent random sequence, which is suitable for information encryption.

**Figure 1:**
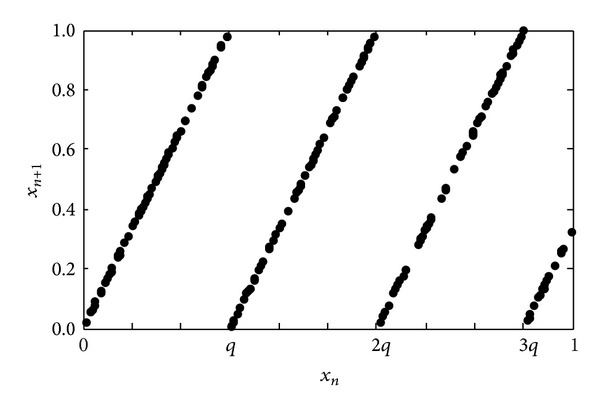
The MPWLCM proposed by this paper.

Based on the PWLCM, we propose an improved piecewise linear chaotic map (MPWLCM) model, which can be denoted by (2) (see also Figure 1):

(2) $$\begin{array}{c} x_{n+1}=F\left(x_{n},q\right)=\frac{x_{n}-\left\lfloor x_{n}/q\right\rfloor \times q}{q}, \end{array}$$

where *q* is the control parameter and 0 < *q* < 0.5. ⌊*x*⌋ denote the maximal integer less than or equal to *x*.

**Figure 2:**
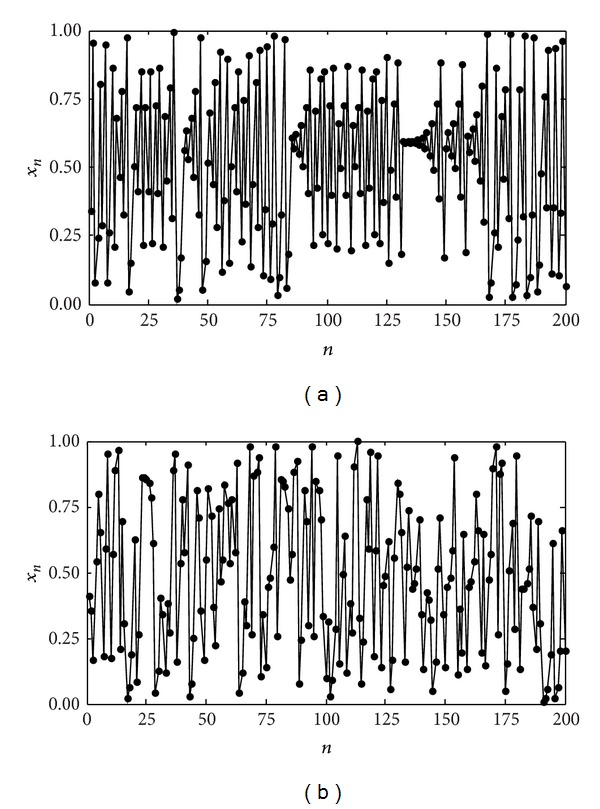
The state sequences of PWLCM and MPWLCM. (a) PWLCM (*q* = 0.3) and (b) MPWLCM (*q* = 0.3).

Figures 2(a) and 2(b) show the state sequences of PWLCM and MPWLCM, respectively. From Figures 2(a) and 2(b), one can see that the sequence of MPWLCM has better performance in randomness than MPWLCM. Hence, MPWLCM is more suitable for information encryption.

## 3. The Proposed Cryptosystem

For a 256-gray-scale image of size *L* = *M* × *N*, it is an integer matrix of *M* rows *N* columns, in which the values range from 0 to 255. Its data can be treated as a one-dimensional vector **P** = {*p*(1), *p*(2), …, *p*(*L*)}, where *p*(*i*) denotes the gray level of the image pixel at ceil (*i*/*N*) row and [ceil(*i*/*N*)−(*i* − 1) × *N*] column.

### 3.1. Generating Permutation Sequence

Given *x*_0_ and *p*, we firstly generate permutation sequence to change the position of image pixel. Different from most proposed methods, our scheme is based on ergodic matrix instead of sorting chaotic sequence. Suppose that **T** = {*t*(1), *t*(2), …, *t*(*L*)} is an ergodic matrix of size 1 × *L*, where *t*(*i*) are integers, *t*(*i*)∈[1, *L*], and *t*(*i*) ≠ *t*(*j*) if *i* ≠ *j*. Our scheme takes the following steps.

*Step  1*. Iterate the PWLCM *x*_*i*+1_ = *F*(*x*_*i*_) by using (2) for *N*_0_ times to get rid of transient effect, where *N*_0_ is a constant; set a one-dimensional matrix flag, which length is *L* and each of its elements is zero; initialize the permutation sequence **T** = {*t*(1), *t*(2), …, *t*(*L*)} : **T** = flag.

*Step  2*. Let *i* ← 1.

*Step  3*. To iterate the PWLCM to obtain a new *x*, compute a integer *j* by using current *x* according to the following formula:

(3) $$\begin{array}{c} j=\mathrm{mod}\left(\text{floor}\left(x\times 10^{15}\right),L\right)+1. \end{array}$$

*Step  4*. Checking the values *j* and flag(*j*), if (*j* = = *i*), or (flag(*j*) = = 1) then repeat Step 3; else then go to Step 5.

*Step  5*. flag(*j*) ← 1; *t*(*i*) ← *j*.

*Step  6*. Let *i* ← *i* + 1, return to Step 3 until *i* reaches *L*.

### 3.2. Generating Diffusion Sequence

*Step  1*. Supposing the diffusion sequence is denoted by **K** = {*k*(1), *k*(2), …, *k*(*L*)}, set *k*(*i*) = 0, *i* = 1,2,…, *L*.

*Step  2*. Let *i* ← 1.

*Step  3*. To iterate the PWLCM to obtain a new *x*, compute *k*(*i*) by using current *x* according to the following formula:

(4) $$\begin{array}{c} k\left(i\right)=\mathrm{mod}\left(\left\lfloor \left(x\times 10^{2}-\left\lfloor x\times 10^{2}\right\rfloor \right)\times 10^{3}\right\rfloor ,256\right). \end{array}$$

*Step  4*. Checking *k*(*i*), if *k*(*i*) < 3, then *k*(*i*) = *k*(*i*) + 3.

*Step  5*. Let *i* ← *i* + 1, return to Step 3 until *i* reaches *L*.

### 3.3. Encryption Algorithm

**Figure 3:**
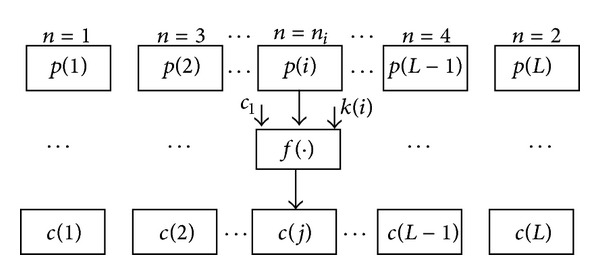
The block diagram of the encryption algorithm.

The encryption process uses the permutation sequence **T** to shuffle the positions of image pixels and uses the diffusion sequence **K** to diffuse the values of image pixels simultaneously. Namely, permutation process will move the pixel of position *i* in plain image to the position *j* in cipher image, where *j* = *t*(*i*). At the same time, the pixel value of position *i* in plain image is altered by using diffusion key *k*(*i*) and the previous encrypted pixel value. Different from most of usual algorithms, the process order *n* is not equivalent to the pixel index *i*, but it is from beginning or end alternately. Namely, (*n* = 1,  *i* = 1), (*n* = 2, *i* = *L*), (*n* = 3, *i* = 2),…, (*n* = *L*, *i* = *L*/2 + 1). If *n* is even, then the processed pixel index is *i* = ceil(*n*/2). At other times the processed pixel index is *i* = (*L* − *n*/2 + 1).  Figure 3 illustrates the block diagram of the proposed encryption algorithm.

The permutation and diffusion process may repeat *R* rounds (*r* = 1 to *R*, *R* ≥ 1). In the first round (*r* = 1), *p*(*i*) denotes the *i*th pixel in original plain image and *c*(*j*) denotes the *j*th pixel in current ciphered image (*i*, *j* = 1,2,…, *L*), where *c*_1_ is the previously outputted cipher pixel value. For *n* = 1, *c*_1_ is equal to a presetting value *c*_0_. In the *r*th round (*r* = 2,3,…, *R*), *p*(*i*) denotes the *i*th pixel in the ciphered image outputted in the (*r* − 1)th turn and *c*(*j*) denotes the *j*th pixel in current ciphered image (*i*, *j* = 1,2,…, *L*). *c*_1_ is the previously outputted cipher pixel value. For *n* = 1, *c*_1_ is the last outputted cipher pixel value in the previous round, where *f*(·) denotes the nonlinear encryption function. The encryption formulas in our scheme are as follows:

(5a) $$\begin{array}{c} c\left(j\right)=\mathrm{mod}\left(p\left(i\right)+c_{1},256\right)⊕k\left(i\right).\;\text{If}\;r=1. \end{array}$$

(5b) $$\begin{array}{c} c\left(j\right)=\mathrm{mod}\left(c\left(i\right)+c_{1},256\right)⊕k\left(i\right).\;\text{If}\;r>1, \end{array}$$

where *i* = 1,2,…, *L* and *j* = 1, 2,…, *L*.

Our encryption algorithm takes the following steps.

*Step  1*. Let *n* ← 1.

*Step  2*. If *n* is even, then *i* ← ceil(*n*/2); otherwise *i* ← (*L* − *n*/2 + 1).

*Step  3*. Obtain *j* by using the permutation sequence **T** : *j* = *t*(*i*).

*Step  4*. Use (5a) or (5b) to permute and diffuse the current pixel simultaneously.

*Step  5*. Let *n* ← *n* + 1.

*Step  6*. If *n* < *L*, then return to Step 1; otherwise one round encryption is complete.

## 4. Experimental Results and Security Analysis

In our experiments, The images for testing are the 256 × 256 traditional images with 8-bit grayscale. The system parameter and initial state of MPWLCM are *q* = 0.3 and *x*_0_ = 0.27. *N*_0_ = 200, *c*_0_ = 150, and *R* = 2.

### 4.1. Key Space Analysis

Key space size is the total number of different keys which can be used in the encryption process. In the proposed algorithm, the secret keys set SK = {*x*_0_, *q*, *N*_0_, *c*_0_}, where *x*_0_ and *q* are double-precision numbers, *c*_0_ is a constant integer and *c*_0_ ∈ [1, 255], and *N*_0_ is a integer. If the computational precision of *x*_0_ and *q* is 10^−16^, then *N*_0_ ∈ [1, 1000]. Therefore, the key space is bigger than 10^16^ × 10^16^ × 255 × 1000, which is much larger than 2^124^. So the encryption algorithm has a large enough key space to resist all kinds of brute-force attacks.

### 4.2. Statistical Analysis

Shannon suggested two methods of diffusion and confusion for frustrating the powerful statistical analysis. Here, we demonstrated the confusion and diffusion properties of our MPWLCM chaotic encryption system. This is shown by a test on the histogram and the correlations of adjacent pixels in the cipher image.

**Figure 4:**
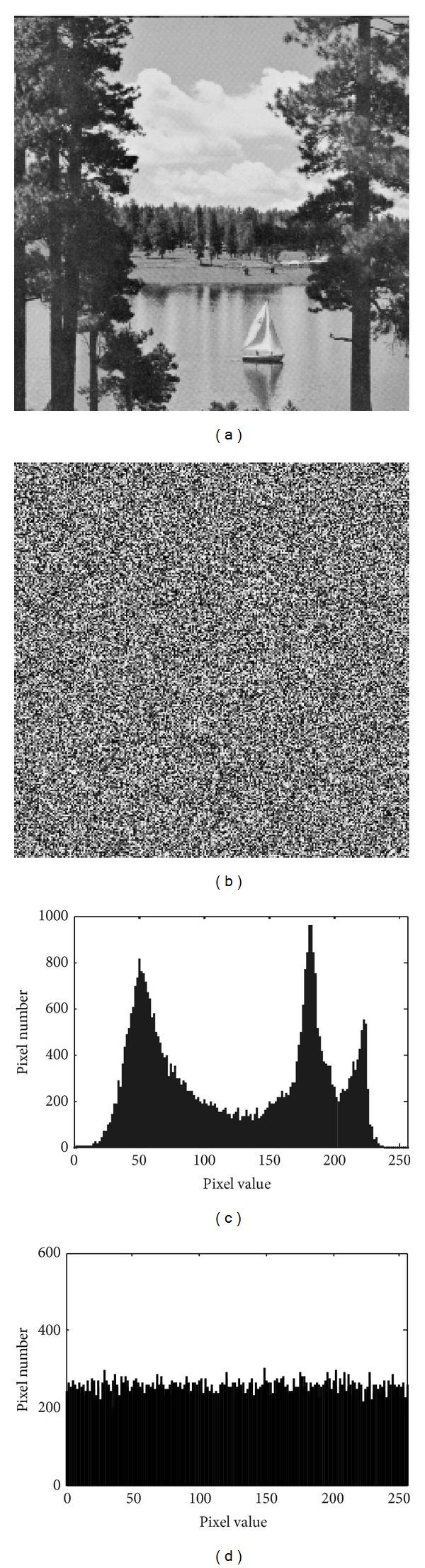
Histogram of the plain image and the cipher image. (a) Plain Lena, (b) encrypted Lena, (c) histogram of plain Lena, (d) histogram of encrypted Lena.

*Histograms of Encrypted Images*. Select several 256 gray-level images with size of 256 × 256 which have different contents and calculate their histograms. One typical example (Sailboat) among them is shown in Figure 4. From Figure 4, we can see that the histogram of the cipher image is fairly uniformed and is significantly different from that of the original image.

*(2) Correlation Coefficients of two adjacent pixels*. To test the correlation between two adjacent pixels in plain image and cipher image, all pairs of two-adjacent pixels (in vertical, horizontal, and diagonal direction) from plain image and cipher image were selected and the correlation coefficients were calculated by using the following formulas:

(6) $$\begin{array}{c} E\left(x\right)=\frac{1}{L}\overset{L}{\underset{i=1}{\sum }}x_{i},\\ D\left(x\right)=\frac{1}{L}\overset{L}{\underset{i=1}{\sum }}{\left[x_{i}-E\left(x\right)\right]}^{2},\\ \text{Conv}\left(x,y\right)=\frac{1}{L}\overset{L}{\underset{i=1}{\sum }}\left[x_{i}-E\left(x\right)\right]\left[y_{i}-E\left(y\right)\right],\\ \gamma_{xy}=\frac{\text{Conv}\left(x,y\right)}{\sqrt{D\left(x\right)}\sqrt{D\left(y\right)}}, \end{array}$$

where *x* and *y* are gray-scale values of two-adjacent pixels in the image and *γ*_*xy*_ is the correlation coefficient of two adjacent pixels. The test results are shown in Table 1. It also shows the results of the existing algorithms in [14]. From Table 1, it can be seen that the encryption scheme satisfies zero cocorrelation, which is of high-level security. Compared with the algorithms proposed by [14], it shows superior performance.

### 4.3. Information Entropy Analysis

Information entropy is the most important feature of randomness. Let *s* be the information source, and the formula for calculating information entropy is

(7) $$\begin{array}{c} H\left(s\right)=-\overset{2^{n}-1}{\underset{i=0}{\sum }}P\left(s_{i}\right)\text{lo}{\text{g}}_{2}\left[P\left(s_{i}\right)\right], \end{array}$$

where *P*(*s*_*i*_) denotes the probability of symbol *s*_*i*_ and 2^*n*^ is the total states of the information source. For a true random source emitting 2^*n*^ symbols, the entropy should be *n*. Take a 256-gray-scale image, for example, and the pixel data have 2^8^ possible values, so the ideal entropy of a 256-gray-scale image must be 8. The information entropy of the cipher-images is shown in Table 2. The obtained values are very close to the theoretical value 8.

### 4.4. Differential Attack

Generally speaking, an opponent may make a slight change (e.g., modify only one pixel) of the encrypted image to observe the change in the result. In this way, we may be able to find out a meaningful relationship between the plain image and the cipher image. This is known as the differential attack. However, if one minor change in the plain image can cause a significant change in the cipher image, with respect to diffusion and confusion, then the differential attack would become very inefficient and useless.

The proposed cryptosystem can ensure two ciphered images different completely, even if there is only one bit difference between plain images. We have done differential analysis by calculating the NPCR (net pixel change rate) and UACI (unified average changing intensity) for several images. Here are the formulas:

(8) $$\begin{array}{c} \text{NPCR}=\frac{\sum_{i,j}D\left(i,j\right)}{M_{1}\times M_{2}}\times 100\%,\\ \text{UACI}=\frac{1}{M_{1}\times M_{2}}\underset{i,j}{\sum }\frac{\left|c_{1}\left(i,j\right)-c_{2}\left(i,j\right)\right|}{255}\times 100\%, \end{array}$$

where *D*(*i*, *j*) represents the difference between *c*_1_(*i*, *j*) and *c*_2_(*i*, *j*). If *c*_1_(*i*, *j*) = *c*_2_(*i*, *j*), then *D*(*i*, *j*) = 0; otherwise *D*(*i*, *j*) = 1. For an 8-bit gray image, the expected estimates are NPCR_E_ = 99.6094% and UACI_E_ = 33.4635%.

**Figure 5:**
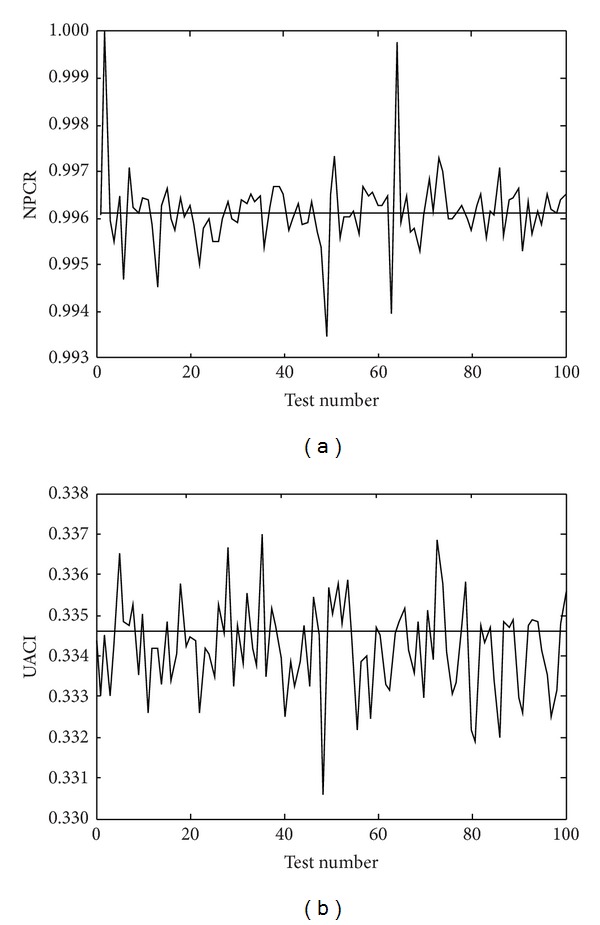
(a) NPCR for 100 modified plain-image Cameraman; (b) UACI for 100 modified plain-image Cameraman.

We have done plaintext sensitivity analysis (differential analysis) by calculating the NPCR and UACI for plain-image Lena, Sailboat, Pepper, and Cameraman. In particular, we have randomly chosen 100 different pixels (one at a time, including the very first and very last pixels of the image) in each plain-image and changed their values slightly, and then we have computed NPCR and UACI for all the cases using (8). The results of NPCR and UACI for the plain-image Cameraman are shown in Figures 5(a) and 5(b), respectively. It is clear that the NPCR and UACI values remain in the vicinity of the expected values (shown by the horizontal lines); that is, the proposed image encryption technique shows extreme sensitivity on the plaintext. Also, Table 3 shows the average values of NPCR and UACI for the plain-image Lena, Sailboat, Pepper, and Cameraman. We can find that the mean NPCR is over 99% and the mean UACI is over 33%. The results show that the proposed algorithm is very sensitive to tiny changes in the plain image; even if there is only one bit difference between two plain images, the encrypted images will be different completely. Thus, the algorithm is robust against differential attack.

**Figure 6:**
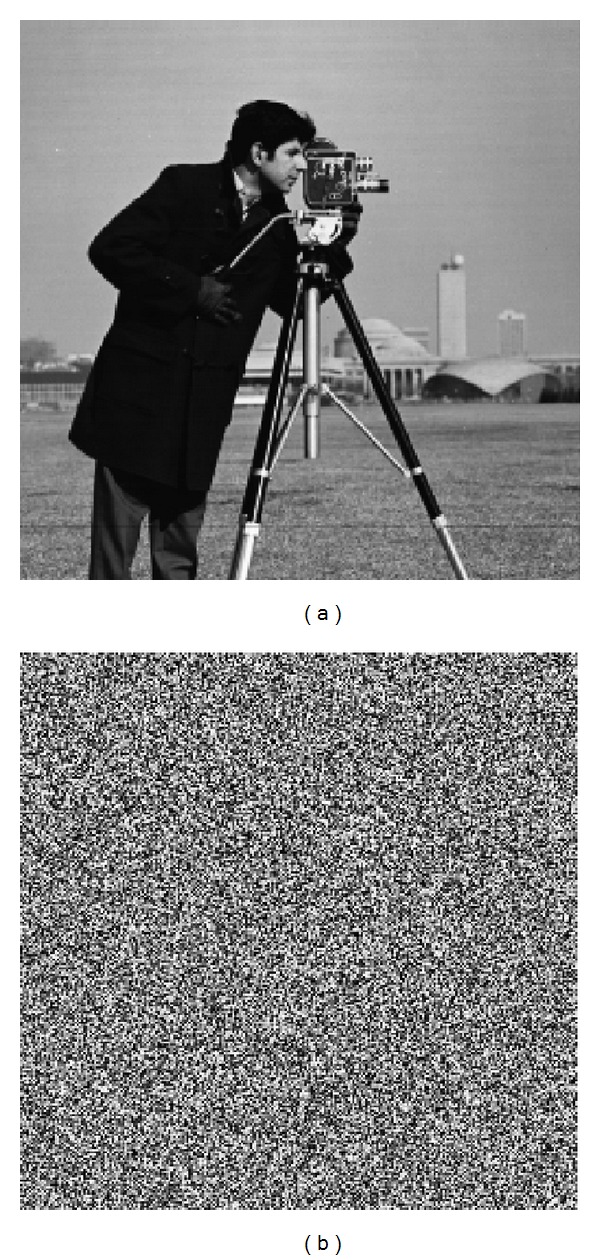
Sensitivity to the secret keys. (a) Cameraman decrypted by correct keys; (b) Cameraman decrypted by wrong keys.

A good encryption algorithm should also be sensitive to the secret keys. For the MPWLCM system, the sensitivity to *x*_0_ and *q* is considered as 10^−16^; Figure 6 shows the sensitivity to the key.  Figure 6(a) is the decrypted Cameraman by correct key (*x*_0_ = 0.27) and Figure 6(b) is the decrypted Cameraman by the wrong key (*x*_0_ = 0.27 + 10^−16^) with a tiny change (10^−16^).

## 5. Conclusion

This paper proposes a symmetric cryptographic system using MPWLCM chaotic system to encrypt grayscale image. We can see that the proposed cryptosystem can process any size of image. Security analysis and experimental results demonstrated the effectiveness of the proposed scheme. The key space is large enough to resist brute-force attacks. Statistical analysis shows that the scheme can well protect the image from the statistical attack. The scheme possesses high sensitivity to plain image and key, so it has a good ability to resist differential attack. With high-level security, it can be used in secure image communications.

## Figures and Tables

**Table 1:** Correlation coefficients of two adjacent pixels in the plain and ciphered images.

Correlation	Horizontal	Vertical	Diagonal
Lena	0.924879	0.959276	0.902644
Encrypted Lena	0.003503	0.000213	0.000728
Encrypted Lena [14]	0.032107	0.027188	0.038393
Sailboat	0.940113	0.936439	0.905541
Encrypted Sailboat	−0.001751	−0.005422	−0.001061
Encrypted Sailboat [14]	0.032347	0.011135	0.014651
Pepper	0.942848	0.945174	0.897210
Encrypted Pepper	−0.000182	0.000357	0.004215
Encrypted Pepper [14]	0.014260	−0.00820751	0.063500
Cameraman	0.933475	0.959223	0.908663
Encrypted Cameraman	−0.000090	−0.007362	0.003039

**Table 2:** Information entropy of the cipher images.

Test image	Information entropy
Lena	7.9976
Sailboat	7.9972
Pepper	7.9972
Cameraman	7.9972

**Table 3:** The mean NPCR and UACI of ciphered images with one bit difference between the plain images.

Image	NPCR (%)	UACI (%)
Lena	99.7421	33.5278
Sailboat	99.7259	33.4237
Pepper	99.7289	33.3548
Cameraman	99.6150	33.4212
